# Rapid-Optimized Process Parameters of 1080 Carbon Steel Additively Manufactured via Laser Powder Bed Fusion on High-Throughput Mechanical Property Testing

**DOI:** 10.3390/ma18153705

**Published:** 2025-08-06

**Authors:** Jianyu Feng, Meiling Jiang, Guoliang Huang, Xudong Wu, Ke Huang

**Affiliations:** 1 College of Pittsburgh, Sichuan University, Chengdu 610200, China; fengjianyu@stu.scu.edu.cn; 2 College of Materials Science & Engineering, Sichuan University, Chengdu 610065, China; jml057589@163.com (M.J.); hgl_sugar@163.com (G.H.); m17311044998@163.com (X.W.); 3 Key Laboratory of Advanced Special Materials & Technology, Ministry of Education, Chengdu 610065, China

**Keywords:** laser powder bed fusion, high-throughput mechanical testing, response surface methodology, 1080 carbon steel

## Abstract

To ensure the sustainability of alloy-based strategies, both compositional design and processing routes must be simplified. Metal additive manufacturing (AM), with its exceptionally rapid, non-equilibrium solidification, offers a unique platform to produce tailored microstructures in simple alloys that deliver superior mechanical properties. In this study, we employ laser powder bed fusion (LPBF) to fabricate 1080 plain carbon steel, a binary alloy comprising only iron and carbon. Deviating from conventional process optimization focusing primarily on density, we optimize LPBF parameters for mechanical performance. We systematically varied key parameters (laser power and scan speed) to produce batches of tensile specimens, which were then evaluated on a high-throughput mechanical testing platform (HTP). Using response surface methodology (RSM), we developed predictive models correlating these parameters with yield strength (YS) and elongation. The RSM models identified optimal and suboptimal parameter sets. Specimens printed under the predicted optimal conditions achieved YS of 1543.5 MPa and elongation of 7.58%, closely matching RSM predictions (1595.3 MPa and 8.32%) with deviations of −3.25% and −8.89% for YS and elongation, respectively, thus validating model accuracy. Comprehensive microstructural characterization, including metallographic analysis and fracture surface examination, revealed the microstructural origins of performance differences and the underlying strengthening mechanisms. This methodology enables rapid evaluation and optimization of LPBF parameters for 1080 carbon steel and can be generalized as an efficient framework for robust LPBF process development.

## 1. Introduction

1080 carbon steel has long been recognized as a critically important engineering material due to its exceptional hardness and wear resistance [1,2]. It finds widespread application in machining, tool manufacturing, automotive components, and related industries [3]. For instance, taps and reamers used in machining leverage the high hardness of 1080 steel to ensure cutting precision and tool longevity [4,5]. Similarly, springs in automotive suspension systems rely on its excellent elasticity and fatigue resistance to maintain stable operation under repeated compressive and tensile loading [3].

Despite its advantages, the production of 1080 plain carbon steel suffers from fundamental limitations due to challenges in precise process control and resultant microstructural heterogeneity. These issues impede its ability to satisfy modern industrial requirements for compositional accuracy, performance stability, environmental sustainability, and cost efficiency. Consequently, achieving the optimal combination of high strength, hardness, and wear resistance typically requires complex, multi-step processing strategies, such as advanced heat-treatment sequences and specialized welding protocols, that have been employed in prior research to finely tailor the steel’s microstructure. Mishra et al. [6] employed a typical cyclic heat treatment to achieve notably high strength (UTS = 2016 MPa) and reasonable ductility (elongation = 11%); Fujii et al. [7] successfully enhanced the toughness and ductility of AISI 1080 steel using liquid CO_2_ during friction stir welding; and Mishra et al. [8] simultaneously improved both the plasticity and strength of AISI 1080 steel via cyclic heat treatment.

In recent years, laser powder bed fusion (LPBF), a core metal additive manufacturing (AM) technology, has demonstrated significant advantages in the efficient, near-net-shape fabrication of complex and precision metal components [9,10,11]. Based on the principle of discrete deposition and layer-by-layer stacking, LPBF utilizes a high-energy laser beam to selectively melt metal powder according to a predefined 3D model path. Crucially, the inherently high cooling rates associated with LPBF promote rapid solidification of the molten metal within the melt pool and produce tailored microstructures in simple alloys that deliver superior mechanical properties [11,12,13]. The core of the LPBF process lies in the precise control of key parameters, such as laser power, scan speed, hatch spacing, and layer thickness, to regulate heating/cooling rates, melt pool morphology, dimensions, overall thermal history, etc. This control ultimately dictates the final part’s density, microstructure, and resultant mechanical properties [14,15,16,17]. Azizi et al. [18] achieved high relative density and optimal surface roughness in Cu–Cr–Zr alloy through systematic LPBF process parameter optimization. Fereiduni et al. [19] fabricated CuNi_2_SiCr alloy via LPBF and investigated the influence of process parameters on its thermal conductivity. Huang et al. [20] systematically investigated the regulation of fracture toughness and fatigue crack propagation in LPBF-fabricated tantalum through process parameter modulation. Lin et al. [21] investigated the effects of process parameters on grain boundary characteristics and crack formation mechanisms during LPBF of nickel-based superalloys.

Minor deviations in LPBF parameter combinations can readily introduce defects such as porosity and microcracks or engender substantial microstructural variations (e.g., grain size, morphology, phase composition). These imperfections markedly degrade dimensional accuracy, density, and—critically—the reliability and consistency of mechanical properties including strength, ductility, and toughness. Such sensitivity is especially acute in processing-sensitive alloys like high-carbon and high-strength steels, which possess inherently narrow processing windows requiring exceptionally precise parameter control. Therefore, a comprehensive understanding and systematic optimization of LPBF process parameters are essential preconditions for the stable, repeatable manufacture of 1080 carbon steel through the LPBF process.

This study investigates 1080 carbon steel, a material known for its high strength and relatively simple chemical composition. While LPBF parameter optimization is well-established, conventional approaches predominantly focus on achieving high density and minimizing defects. Traditional LPBF process optimization has predominantly focused on achieving high density and reducing defects, with the assumption that high density itself ensures superior mechanical properties. However, in practice, components with density exceeding 99% often exhibit low strength, ductility, or fatigue resistance. This is because mechanical properties are determined by microstructural characteristics such as grain size and dislocation density, rather than density alone. In contrast, this study takes mechanical properties as the core optimization target and constructs a parameter optimization framework to achieve customized performance for the narrow processing window of 1080 steel, filling the gap in existing research. Furthermore, systematic optimization specifically targeting mechanical performance metrics, particularly for processing-sensitive high-carbon steels like 1080 with inherently narrow processing windows, remains less explored. So, the objective is to achieve an optimal balance between yield strength and elongation through precise control of key LPBF process parameters. A systematic variation of laser power and scan speed was employed to fabricate batches of tensile specimens, which were subsequently evaluated using a high-throughput mechanical testing platform (HTP). The RSM method was utilized to develop predictive models that quantitatively correlate processing parameters with mechanical properties, specifically yield strength (YS) and elongation. RSM was further applied to identify optimal and suboptimal parameter sets that maximize mechanical performance and was verified by actual experiments. Comprehensive microstructural characterization, including metallographic analysis and fracture surface examination, elucidated the microstructural origins of the observed mechanical behavior and revealed the underlying strengthening mechanisms. This work presents an efficient and reliable framework for optimizing LPBF processing of high-performance 1080 carbon steel, enabling targeted mechanical property enhancement and offering generalizability to other alloy systems and processes.

## 2. Materials and Experimental Methods

### 2.1. Raw Materials

**Figure 1:**
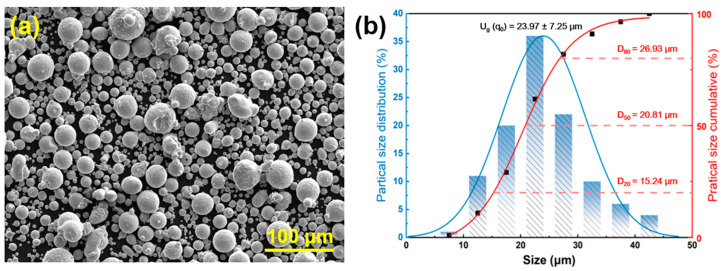
Morphology and size distribution of powder: (**a**) SEM, (**b**) size distribution.

The 1080 carbon steel powder used in this study was supplied by Hebei Chuangying Metal Materials Co., Ltd. (Xingtai, China) and produced via gas atomization. It exhibits a Hall flow rate of 13.2 s per 50 g, with chemical composition detailed in Table 1; the powder particles display predominantly spherical morphology with uniform size distribution. The high sphericity (≥98.5%) and D50 particle size of 20.81 μm render this powder highly suitable for LPBF processing (Figure 1).

### 2.2. Experimental Methods

**Figure 2:**
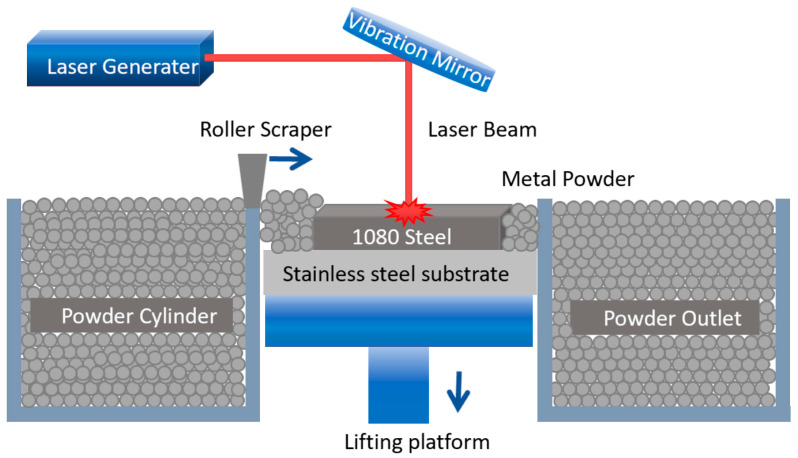
Schematic diagram of LPBF technology.

LPBF processing was performed using an HBD-80 system (Hanbang Union 3D Technology Co., Ltd., Shanghai, China) equipped with a YLR-500 fiber laser (beam diameter: 33 μm; wavelength: 1060 nm). Prior to fabrication, 1080 steel powder was dried for 8 h in an argon-purged desiccator to eliminate residual moisture and prevent pore formation. During printing, continuous argon flow maintained oxygen levels below 5 ppm to suppress interlayer oxidation (Figure 2 illustrates the LPBF scheme). Based on previous research, process parameter development employed the following ranges: laser power (P) = 100–375 W, scan speed (V) = 400–600 mm/s, hatch spacing (H) = 120 μm, and layer thickness (t) = 30 μm. The volumetric energy density (VED), calculated as VED = P/(V·H·t), ranged from 46.3 to 260.4 J/mm^3^, providing a preliminary indicator of energy input. Table 2 details the 16 parameter combinations and corresponding VED values used for specimen fabrication. Conventional mechanical testing approaches would incur prohibitive time and resource costs for this parameter matrix. Consequently, we employed our self-developed high-throughput testing platform [22] to efficiently acquire tensile property data, with three tests conducted per set and the results averaged.

To identify the optimal process parameters for maximizing mechanical performance in high-density 1080 carbon steel, an analytical model based on RSM was established. Laser power (P), scan speed (v), and hatch spacing (h) were selected as the key experimental factors. Utilizing high-throughput mechanical testing data, the Design-Expert 13 software was employed to construct a statistically robust experimental matrix. Yield strength (YS) and elongation (EL) were designated as the response variables to guide optimization. A quadratic polynomial regression model [23] (Equation (1)) was implemented to construct a mathematical relationship approximating the parameter property mapping between experimental factors and response targets.

(1) $$y=\alpha_{0}+\sum_{i=1}^{k}\alpha_{i}x_{i}+\sum_{i=1}^{k}\alpha_{ii}x_{i}^{2}+\sum_{i<j}^{k}\alpha_{ij}x_{i}x_{j}+\omega$$

where y represents the response variable, x represents the predictor variable, k represents the number of experimental factors, α_0_ is the constant term, α_i_ is the coefficient of the first-order term, α_ii_ is the coefficient of the second-order term, α_ij_ is the coefficient of the interaction term, and w represents the random error.

### 2.3. Material Characterization Techniques

Sample density was measured via Archimedes’ principle using a MAY-D80 automated density balance (MAYZUM Scientific Instruments (Shenzhen) Co., Ltd., Shenzhen, China). Metallographic examination of the macrostructure was performed with a Zeiss Axio Imager 40MAT optical microscope (Carl Zeiss (China) Co., Ltd., Suzhou, China). For electron backscatter diffraction (EBSD) analysis, samples underwent sequential preparation: electrochemical polishing in 10% HClO_4_ ethanol solution, followed by broad-beam ion milling using a Gatan PECS II 685 system. EBSD orientation maps were then acquired on a Zeiss Gemini 300 scanning electron microscope Carl Zeiss AG, Oberkochen, Germany) equipped with an Oxford Instruments Symmetry EBSD detector. All EBSD data were processed using AZtecCrystal software (v2.1).

## 3. Results

### 3.1. Mechanical Properties from High-Throughput Tensile Testing

**Figure 3:**
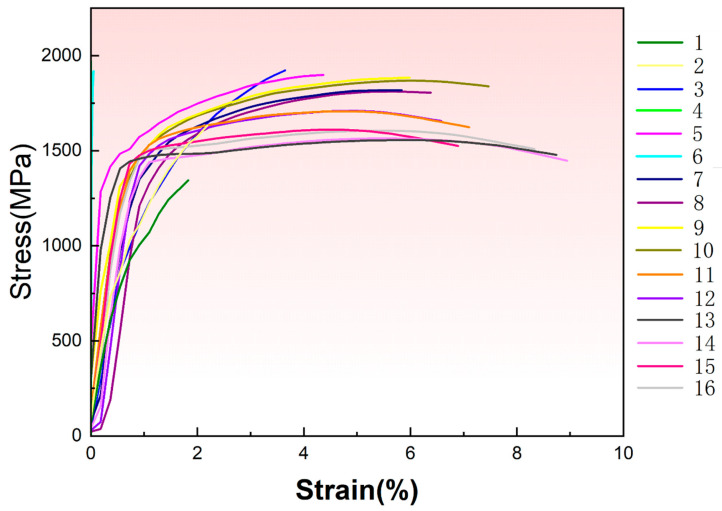
Stress–strain curves of 1080 specimens through high-throughput tensile testing.

Strength and ductility, as critical metrics for assessing mechanical performance, are often significantly enhanced in LPBF-fabricated components due to rapid solidification and thermal cycling effects. Leveraging our self-developed high-throughput tensile platform with automated parallel testing capability, we efficiently evaluated these properties. The corresponding stress–strain curves are presented in Figure 3, while key mechanical property data are summarized in Table 3.

### 3.2. Process-Property Analytical Model

**Figure 4:**
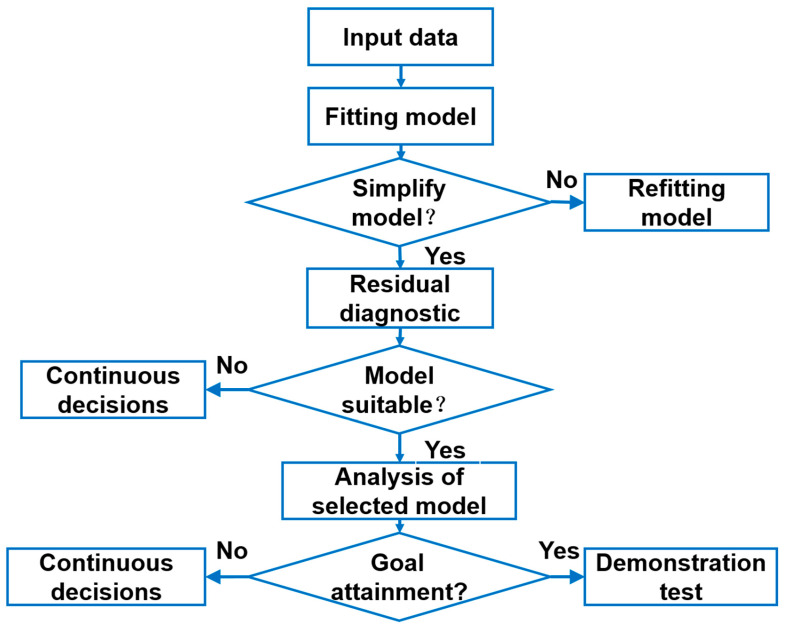
RSM parameter optimization process [17].

Leveraging the tensile performance dataset, we employed response surface methodology (RSM) via Design-Expert 13 software to establish predictive relationships between process parameters and mechanical properties. Figure 4 (adapted from [24]) illustrates the RSM optimization workflow. Regression analysis revealed quadratic polynomial models provided the best fit for the experimental data. The two-variable polynomial equations relating yield strength (YS) and elongation (EL) to laser power (P) and scan speed (V) are expressed as follows:

YS = +3271.77605 − 5.88014 × P − 3.72528 × V + 0.012212 × P × V(2)

EL = 5.05 + 2.85 × P + 0.0515 × V − 0.0255 × P × V − 0.9211 × P^2^ (3)

**Figure 5:**
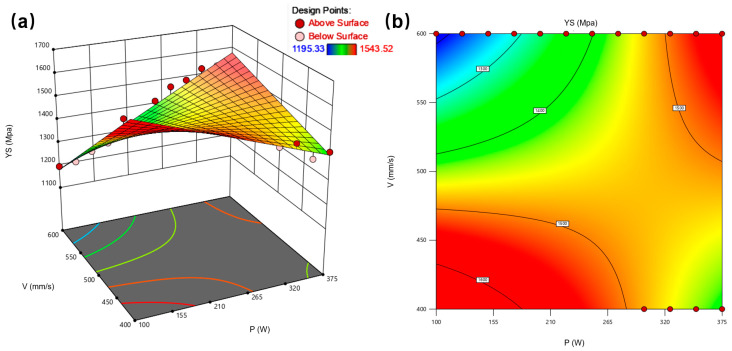
Influence of laser power and laser velocity on yield stress of LPBF 108: (**a**) 3D surface; (**b**) contour maps illustrate the effect of power and velocity on the YS.

Figure 5a presents the 3D response surface for yield strength (YS), revealing a nonlinear relationship between YS and the laser power/scan speed parameters. The response surface exhibits a distinct maximum (red region) corresponding to peak YS values and a minimum (blue region) associated with lower strength. Figure 5b displays the corresponding contour plot, demonstrating the combined effect of laser power and scan speed on YS. Analysis indicates the optimal parameter window for maximizing yield strength occurs within P ≈ 250–350 W and V ≈ 500–550 mm/s, where contour lines converge toward the response maximum.

**Figure 6:**
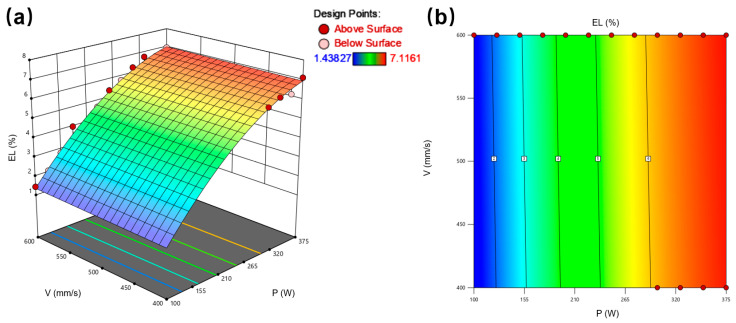
Influence of laser power and laser velocity on elongation of LPBF 1080: (**a**) 3D surface; (**b**) contour maps illustrate the effect of power and velocity on the EL.

Figure 6a presents the 3D response surface for elongation (EL), demonstrating a positive correlation with increasing laser power and decreasing scan speed. The monotonic ascending trend indicates enhanced material ductility at higher energy input regimes. Figure 6b displays the corresponding contour plot, revealing a region of maximum elongation (red contours) concentrated at high laser power and low scan speed combinations. This parameter space corresponds to volumetric energy densities exceeding 180 J/mm^3^, where improved inter-track fusion reduces void formation and enhances plastic deformation capacity.

Table 4 and Table 5 present the analysis of variance (ANOVA) results for the yield strength and elongation regression models. We employed ANOVA to evaluate model significance and confidence levels [25], where the F-statistic and *p*-value determined the statistical significance of model terms [26,27]. The F-statistic quantifies the ratio of model variance to residual variance, with higher values indicating greater model significance. The *p*-value represents the probability of obtaining an F-statistic at least as extreme under the null hypothesis, with *p* < 0.05 denoting statistical significance and *p* < 0.01 indicating high significance [28]. Larger *p*-values corresponded to increased probability of model error. Furthermore, ANOVA revealed comparable influence magnitudes of laser power (P) and scan speed (V) on yield strength and elongation, with all linear and quadratic terms exhibiting *p* < 0.01.

Table 6 presents comprehensive model reliability metrics. The coefficient of determination (R^2^) and adjusted R^2^ (adj-R^2^) approach unity (0.9602/0.9503 for YS; 0.9896/0.9859 for EL), confirming exceptional agreement between experimental and predicted values [28]. A coefficient of variation (C.V.) of 1.90% for YS and 4.17% for EL indicates minimal data dispersion, while adequate precision ratios (YS: 30.0679; EL: 46.6148) substantially exceed the recommended threshold of 4. These statistically robust validation metrics demonstrate the model’s efficacy for predicting mechanical properties in LPBF-processed components.

**Figure 7:**
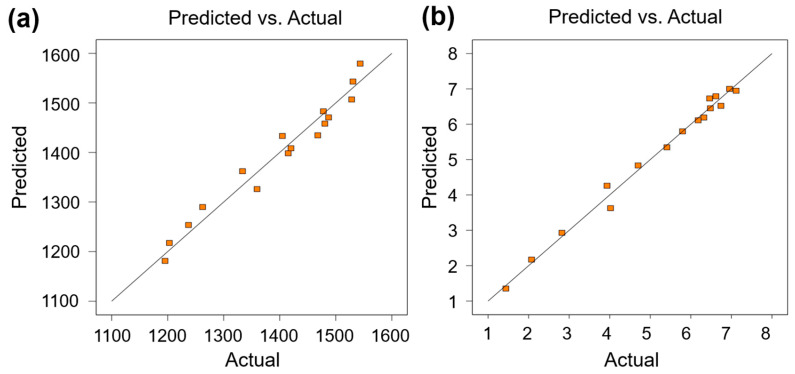
Comparison of predicted and actual values of (**a**) yield stress and (**b**) EL of LPBF 1080 steel.

To evaluate model fidelity and predictive accuracy, we conducted comparative analysis between experimental measurements and model-predicted values. Figure 7 presents the experimental vs. predicted plots for yield strength (YS) and elongation (EL) in LPBF-processed 1080 carbon steel. Data points demonstrate tight clustering about the parity line with minimal deviation. This alignment validates the model’s robustness and high predictive precision within the investigated parameter space.

### 3.3. Process Optimization and Verification

**Figure 8:**
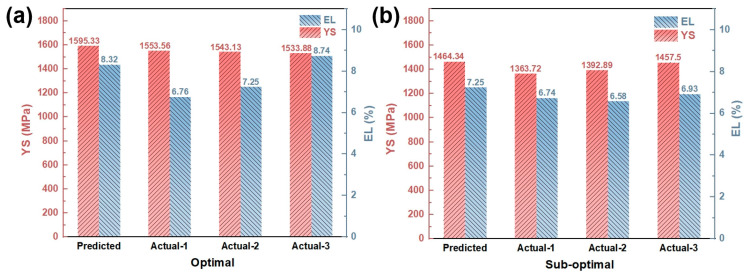
Comparison of predicted and actual values of yield stress and EL of LPBF 1080 steel: (**a**) optimal; (**b**) sub-optimal.

To validate the optimization methodology, experimental verification was performed using two parameter sets recommended by the RSM software (Design-Expert 13): optimal parameters (P = 375 W, v = 600 mm/s, h = 120 μm) and sub-optimal parameters (P = 350 W, v = 400 mm/s, h = 120 μm), as presented in Table 7 and Figure 8. The deviation rate is defined as the ratio of the actual average value to the predicted value (Table 7), with a prediction deemed reliable when this rate is less than 10% [29]. For yield strength (YS), the deviation rates of the optimal and sub-optimal groups are −3.25% and −4.07%, respectively—both well within the 10% threshold. Analysis of the coefficient of variation (C.V.) further confirms the high repeatability of mechanical test results: the optimal group exhibits a YS C.V. of only 0.52%, while the sub-optimal group shows 2.79%. This close alignment between predicted and experimental YS values underscores the predictive model’s accuracy and reliability. Regarding elongation (EL), the deviation rates for both parameter sets are also below 10% (optimal: −8.89%; sub-optimal: −6.90%), validating the model’s effectiveness even for this property, which is highly sensitive to minor surface scratches, internal microcracks, or localized residual stress concentrations. These factors can act as initiation sites for deformation localization under tensile loading; however, the model demonstrates robust predictive capability despite such sensitivities.

**Figure 9:**
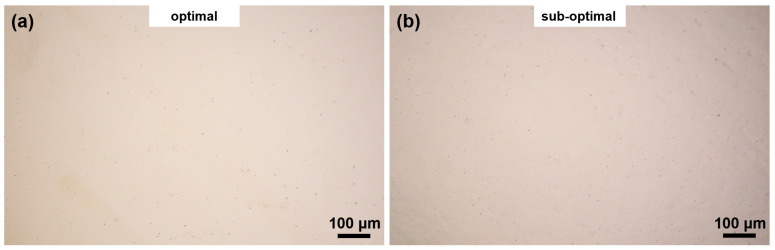
Metallographic micrographs of 1080 steel: (**a**) optimal, (**b**) sub-optimal.

Crucially, metallographic analysis (Figure 9) demonstrates near-full density (>99.2%) with negligible defect formation in both parameter sets. This microstructural evidence, combined with the model’s predictive accuracy for yield strength, validates the efficacy of our RSM-based approach for optimizing LPBF processing of 1080 carbon steel.

**Figure 10:**
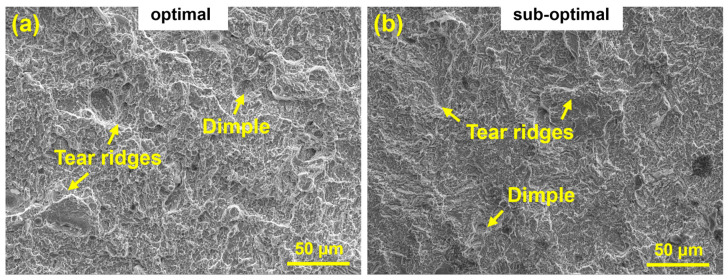
Scanning electron microscope (SEM) images of tensile fracture surfaces: (**a**) optimal, (**b**) sub-optimal.

Figure 10 presents tensile fracture morphologies of specimens fabricated under (a) optimal and (b) sub-optimal parameters. Both exhibit ductility-dominated mixed fracture modes yet display distinct plastic deformation heterogeneity: the sub-optimal specimen (Figure 10b) shows widely dispersed dimples (including elongated variants) with tear ridges, cleavage steps, and river patterns, indicating localized stress concentration and heterogeneous plastic constraints. Conversely, the optimal specimen (Figure 10a) features uniformly distributed equiaxed dimples with narrow size distribution, demonstrating superior energy dissipation during plastic deformation. This morphological contrast directly correlates with differentiated fracture mechanics: the homogeneous dimple structure in Figure 10a creates higher energy barriers against crack propagation, enhancing fracture toughness and elongation. Crucially, neither specimen reveals pores or inclusions, confirming exceptional density that effectively suppresses stress concentration initiation, thereby providing microstructural assurance for enhanced tensile performance.

## 4. Discussion

### 4.1. Optimal vs. Sub-Optimal Parameters: Process–Microstructure–Property Linkages

To elucidate the underlying mechanisms responsible for the performance divergence between optimal and sub-optimal process sets, detailed characterization and analysis were conducted on both specimens.

#### 4.1.1. Grain Size and Morphology

**Figure 11:**
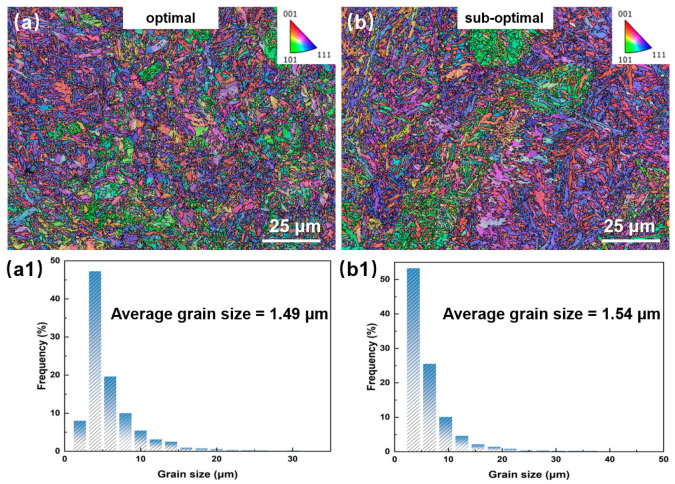
Grain distribution of LPBF- fabricated 1080 specimens during different processes: (**a**,**a1**) optimal specimen; (**b**,**b1**) sub-optimal specimen.

Figure 11 presents the size distribution figures of LPBF-processed 1080 carbon steel fabricated under (a) optimal (P = 375 W, h = 120 μm, v = 600 mm/s, VED = 173.60 J/mm^3^) and (b) sub-optimal (P = 350 W, h = 120 μm, v = 400 mm/s, VED = 243.06 J/mm^3^) parameters. Colors represent distinct crystallographic orientations: red = <001>, green = <101>, and blue = <111>. Quantitative analysis reveals refined grain structures with average sizes of 1.49 μm (Figure 11(a1)) and 1.54 μm (Figure 11(b1)). This grain coarsening at higher VED results from reduced nucleation density, where grain growth dominates over nucleation [28], consistent with observations in Invar alloys [30] and steels [31]. According to the Hall–Petch relationship, the finer-grained optimal specimen exhibits enhanced strength due to heightened grain boundary strengthening.

#### 4.1.2. Crystallographic Texture

**Figure 12:**
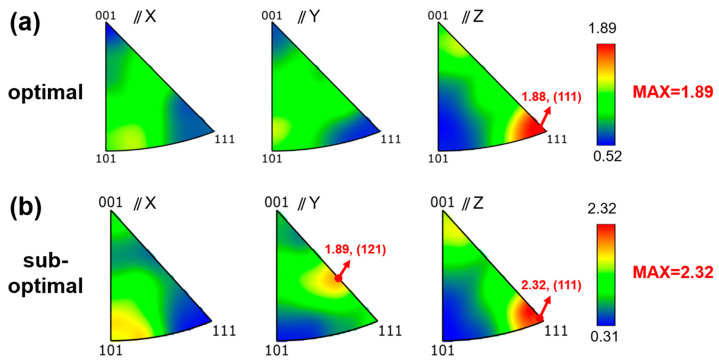
Inverse pole figures (IPFs) of LPBF-fabricated 1080 specimens during different processes: (**a**) optimal specimen; (**b**) sub-optimal specimen.

Figure 12 presents inverse pole figures (IPF) of optimal and sub-optimal specimens, revealing distinct crystallographic texture characteristics. Figure 12a exhibits a strong <111>//Z fiber texture with no pronounced preferential orientations along other axes. In contrast, Figure 12b demonstrates a complex texture comprising the following: strong <111>//Z and <121>//Y components and weaker <111>//X, <101>//Y, and <101>//Z orientations. Quantitative texture analysis confirms significant differences in texture index (1.24 vs. 0.78), demonstrating process-dependent texture evolution in LPBF-processed 1080 steel.

#### 4.1.3. Geometric Dislocation Density

**Figure 13:**
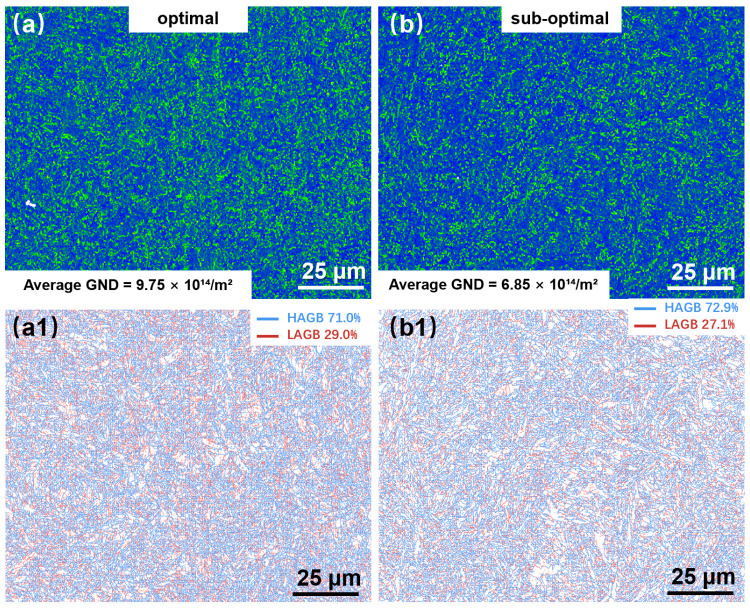
KAM figures (**a**,**b**) and distribution of grain boundary angle (**a1**,**b1**) of LPBF- fabricated 1080 specimens during different processes: (**a**) optimal specimen; (**b**) sub-optimal specimen.

During LPBF processing, rapid cooling induces significant thermal stress accumulation, promoting plastic deformation within the solidified materials [32]. Kernel average misorientation (KAM) quantifies plastic strain magnitude and indicates localized concentrations of thermal stress or defect density [33]. Figure 13 presents KAM maps and grain boundary character distributions. Regions of elevated plastic deformation appear green in Figure 13a,b, corresponding to higher KAM values. Quantitative analysis reveals a greater grain boundary density (9.75 × 10^14^ m^−2^) in the optimal parameter specimen (Figure 13a) vs. 6.85 × 10^14^ m^−2^ in the sub-optimal specimen (Figure 13b), consistent with the finer grain size observed in IPF analysis. Figure 13(a1,b1) displays grain boundary misorientation distributions, with low-angle boundaries (2–15°) marked red and high-angle boundaries (>15°) blue. Increasing VED from 173.6 J/mm^3^ (optimal) to 243.06 J/mm^3^ (sub-optimal) correlates with the decreased low-angle boundary fraction of 29.0% to 27.1% and increased high-angle boundary fraction of 71.0% to 72.9%. This transition suggests enhanced recovery processes at higher energy input.

#### 4.1.4. Process–Structure–Property Linkages of the Optimal and Sub-Optimal Specimens

Variations in key process parameters, such as laser power and scan speed, influence the volumetric energy density (VED), which in turn directly governs the microstructural and mechanical properties evolution of 1080 carbon steel fabricated via LPBF. The optimal parameter set characterized with lower VED of 173.6 J/mm^3^ promotes refined grains (1.49 μm average size) and elevated geometrically necessary dislocation (GND) density (9.75 × 10^14^ m^−2^), with kernel average misorientation (KAM) analysis confirming concentrated plastic deformation zones. Conversely, the sub-optimal set with higher VED of 243.06 J/mm^3^ induces grain coarsening (1.54 μm) and reduced GND density (6.85 × 10^14^ m^−2^). These microstructural distinctions directly modulate mechanical properties through grain boundary and dislocation strengthening mechanisms. The optimal specimen achieves superior yield strength (1543.52 MPa) and elongation (7.58%) vs. the sub-optimal specimen (1404.70 MPa, 6.75%), thereby conducting a rough process–structure–property (P-S-P) linkage paradigm.

### 4.2. Advantages of This High-Throughput Testing and Analytical Modeling Approach

#### 4.2.1. High Efficiency

This process optimization framework achieved completion within 50 h, primarily through three synergistic advancements: Firstly, the high-throughput tensile platform enabled automated batch testing with 10-fold efficiency gains over manual methods. Secondly, it eliminated time-intensive polishing and detailed microstructural characterization for all density-qualified specimens. Thirdly, RSM streamlined data analysis, model construction, and parameter optimization into a unified computational workflow.

#### 4.2.2. Fine-Tuning Process Parameters to Achieve Customized Performance

Conventional process optimization predominantly focuses on densification, defect control, and microstructural evolution, with mechanical properties typically measured only at selected parameter sets. Therefore, a traditional approach fundamentally limits systematic establishment of process-property correlations. In contrast, our methodology leveraging 16 distinct process-property datapoints developed an analytical model capable of recommending optimal process parameters for arbitrary customized performance targets within defined ranges. This data-driven framework holds significant implications for demanding applications such as aerospace and medical components where customized performance is critical.

## 5. Conclusions

This study fabricated 1080 carbon steel using the LPBF technique and proposes a rapid process optimization framework based on high-throughput mechanical testing and response surface methodology (RSM).

Optimal process parameters were determined through response surface methodology (RSM), and the relationship between mechanical properties and microstructure was systematically investigated. The main conclusions are as follows:

(1) This performance-oriented process optimization framework, built upon a high-throughput testing platform and advanced data analysis techniques, demonstrates exceptional efficiency. Within only 50 h, a comprehensive process–property database and predictive model were established, enabling the identification of processing parameters that yield the customized values of yield strength (YS) and elongation (EL).
(2) Under the optimized process conditions, the LPBF-fabricated 1080 carbon steel exhibited a yield strength of 1543.52 MPa and an elongation of 7.58%. These values are in close agreement with the RSM-predicted results (1595.33 MPa and 8.32%), corresponding to relative errors of −3.25% and −8.89%, respectively, thereby validating the accuracy and reliability of the predictive model.
(3) The superior mechanical performance at the optimal condition is primarily attributed to a relatively low volumetric energy density (VED), which results in refined grain structures (average grain size: 1.49 µm), an elevated geometrically necessary dislocation (GND) density (9.75 × 10^14^ m^−2^), and grain boundary-mediated dislocation strengthening. These underlying mechanisms were revealed through detailed microstructural characterization.

## Figures and Tables

**Table 1:** Chemical composition of 1080 plain carbon steel.

Elements	C	Si	Mn	S	P	Fe
Wt%	0.75	0.19	0.79	0.01	0.01	Bal.

**Table 2:** Process parameters of specimens.

No.	P (W)	V (mm/s)	h (μm)	VED (J/mm^3^)
1	100	600	120	46.3
2	125	600	120	57.9
3	150	600	120	69.4
4	175	600	120	81.0
5	200	600	120	92.6
6	225	600	120	104.2
7	250	600	120	115.7
8	275	600	120	127.3
9	300	600	120	138.9
10	325	600	120	150.5
11	350	600	120	162.0
12	375	600	120	173.6
13	350	400	120	243.1
14	375	400	120	260.4
15	300	400	120	208.3
16	325	400	120	225.7

**Table 3:** High-throughput tensile test results.

No.	P (W)	V (mm/s)	h (μm)	Yield Strength (MPa)	Elongation (%)
1	100	600	120	1195.3 ± 5.8	1.4 ± 0.1
2	125	600	120	1203.0 ± 6.6	2.1 ± 0.2
3	150	600	120	1237.2 ± 13.1	2.8 ± 0.5
4	175	600	120	1262.4 ± 17.1	4.0 ± 0.4
5	200	600	120	1359.5 ± 16.7	3.9 ± 0.6
6	225	600	120	1333.6 ± 13.0	4.7 ± 0.1
7	250	600	120	1415.1 ± 20.2	5.4 ± 0.4
8	275	600	120	1467.9 ± 18.7	5.8 ± 0.5
9	300	600	120	1487.2 ± 15.6	6.3 ± 0.2
10	325	600	120	1528.4 ± 12.6	6.7 ± 0.5
11	350	600	120	1530.6 ± 10.9	6.6 ± 0.4
12	375	600	120	1543.5 ± 8.0	7.0 ± 0.1
13	350	400	120	1404.7 ± 19.2	6.5 ± 1.4
14	375	400	120	1420.1 ± 12.9	7.1 ± 0.5
15	300	400	120	1477.8 ± 7.1	6.2 ± 0.8
16	325	400	120	1480.5 ± 15.8	6.5 ± 0.8

**Table 4:** Variance analysis of regression equation terms of YS.

Source	Sum of Squares	df	Mean Square	F-Value	*p*-Value Prob > F	Significant
Model	203,124.9099	3	67,708.3033	96.6238	<0.0001	Significant
P	616.6186	1	616.6186	0.8780	0.3667	
V	7703.0096	1	7703.0097	10.9926	0.0061	
PV	18,012.3838	1	18,012.3838	25.7047	0.0002	
Residual	8408.9021	12	700.74183			
Cor Total	211,533.8119	15				

**Table 5:** Variance analysis of regression equation terms of EL.

Source	Sum of Squares	df	Mean Square	F-Value	*p*-Value Prob > F	Significant
Model	49.31	4	12.33	262.50	<0.0001	Significant
P	4.21	1	4.21	89.70	<0.0001	
V	0.0026	1	0.0026	0.0556	0.8179	
P × V	0.0003	1	0.0003	0.0072	0.9340	
P^2^	1.24	1	1.24	26.43	0.0003	
V^2^	0.0000	0				
Residual	0.5166	11	0.0470			
Cor Total	49.83	15				

**Table 6:** Reliability analysis of regression variance of YS and EL.

	Std. Dev.	Mean	C.V.%	R^2^	Adjusted R^2^	Predicted R^2^	Adeq Precision
YS	26.47	1396.68	1.90	0.9602	0.9503	0.9312	30.0679
EL	0.2167	5.19	4.17	0.9896	0.9859	0.9769	46.6148

**Table 7:** P, V, H of predicted YS-optimal specimen and EL-optimal specimen.

		YS (MPa)	EL (%)
Optimal	Predicted	1595.33	8.32
	Actual-1	1553.56	6.76
	Actual-2	1543.13	7.25
	Actual-3	1533.88	8.74
	Actual Mean	1543.52	7.58
	Actual STD	8.04	0.84
	Actual C. V	0.52%	11.1%
	Deviation rate	−3.25%	−8.89%
		YS (MPa)	EL (%)
Sub-optimal	Predicted	1464.34	7.25
	Actual-1	1363.72	6.74
	Actual-2	1392.89	6.58
	Actual-3	1457.5	6.93
	Actual Mean	1404.70	6.75
	Actual STD	39.19	0.14
	Actual C. V	2.79%	2.12%
	Deviation rate	−4.07%	−6.90%

## Data Availability

The original contributions presented in this study are included in the article. Further inquiries can be directed to the corresponding authors.
